# Supplementary material for: Oxidative Stress-Induced Alterations of Cellular Localization and Expression of Aquaporin 1 Lead to Defected Water Transport upon Peritoneal Fibrosis
Source: Biomedicines. 2022 Mar 30;10(4):810. doi: 10.3390/biomedicines10040810 (PMC9031283; doi:10.3390/biomedicines10040810)
Supplement: Supplementary file 1 [file biomedicines-10-00810-s001.zip › biomedicines-1635604-Figure S1-S3.pdf]

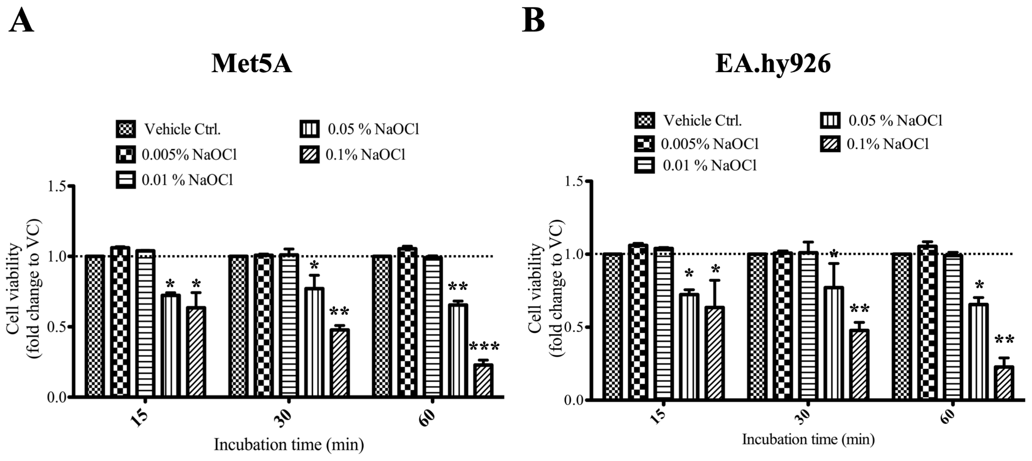

Figure S1. Cytotoxic evaluations of NaClO on MeT5A and EA.hy926, related to figure 4. (A) Cell viability assay was performed to evaluate cytotoxicity of NaClO on mesothelial cell line MeT5A. Significant decrease in cell viability was measured when NaClO concentration was higher than 0.05%. (B) Cell viability assay was performed to evaluate cytotoxicity of NaClO on endothelial cell line EA.hy926. Significant decrease in cell viability was measured when NaClO concentration was higher than 0.05%. (*$p < 0.05$, **$p < 0.01$, ***$p<0.001$). For each test, 3 replicates were performed, fold changes with standard deviation (SD) were presented.

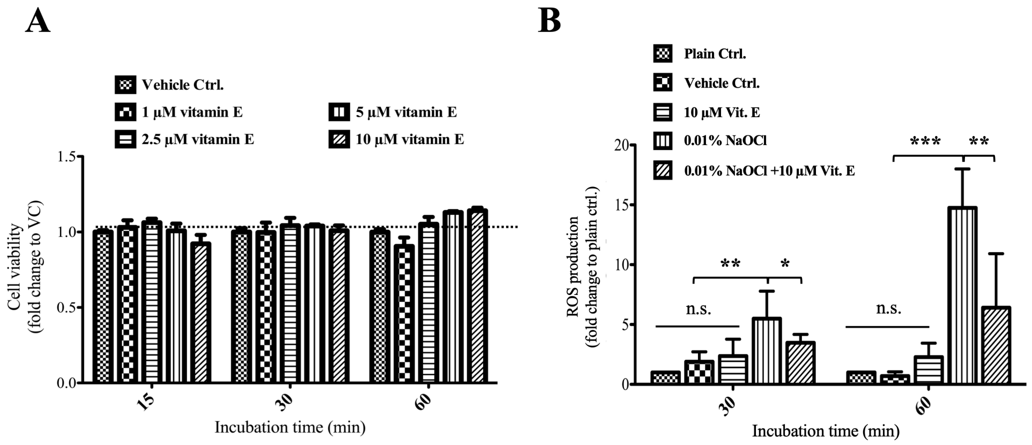

Figure S2. Antioxidant vitamin E significantly reduced NaClO-induced intracellular ROS production, related to figure 4. (A) Cell viability assay was performed to evaluate cytotoxicity of vitamin E on mesothelial cell line MeT5A. No apparent cytotoxicity was measured up to 10 μM of vitamin E. (B) 10 μM of vitamin E significantly reduced NaClO-induced ROS supported NaClO incubation significantly increased intracellular ROS production. (*$p < 0.05$, **$p < 0.01$, ***$p<0.001$). For each test, 3 replicates were performed, fold changes with standard deviation (SD) were presented.

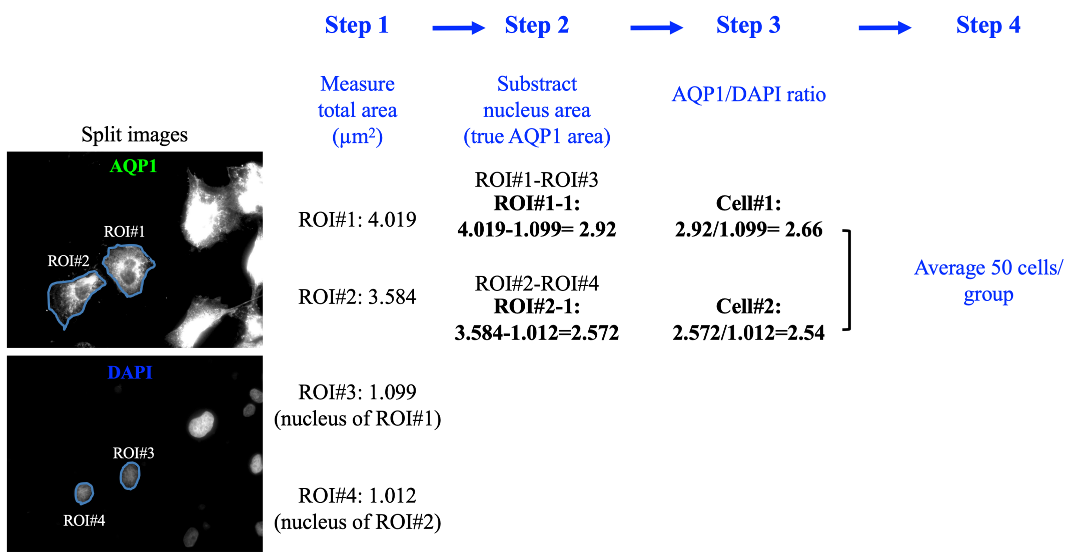

Figure S3. Self-defined membrane distribution assay for intracellular AQP1, related to Figure 6. To measure cellular and membrane distribution of AQP1, a self-defined assay was established by measuring signal area of both AQP1 and cell nucleus (DAPI). Surface area of both AQP1 (region of interest, ROI#1, 2) and cell nucleus (ROI#3, 4) were measured based on visible fluorescent signals (step 1). Subtraction of total AQP1 surface area with nucleus surface area allowed true measurement of intracellular AQP1 distribution (step 2). Ratio between true AQP1 surface area and nucleus area (AQP1/DAPI) indicated the level of AQP1 dispersion from the center of the nucleus suggested either dispersed or aggregated intracellular AQP1 pattern in these cells (step 3).
