# Supplementary material for: Oxidative Stress-Induced Alterations of Cellular Localization and Expression of Aquaporin 1 Lead to Defected Water Transport upon Peritoneal Fibrosis
Source: Biomedicines. 2022 Mar 30;10(4):810. doi: 10.3390/biomedicines10040810 (PMC9031283; doi:10.3390/biomedicines10040810)
Supplement: Supplementary file 1 [file biomedicines-10-00810-s001.zip › biomedicines-1635604-Figure S4.pdf]

Original whole membrane of Western blotting detection in manuscripts.

Whole PVDF membrane from all Western blotting analyses were presented. Target protein signal was indicated with corresponding molecular weight.

- **The following were referred to western blot results in Figure 3**

AQP1

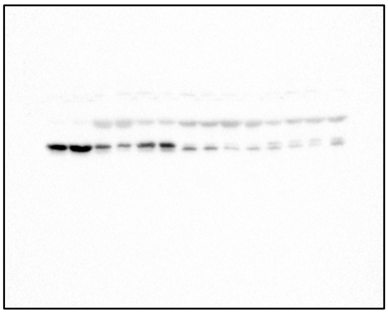

– 28kDa

EEF2

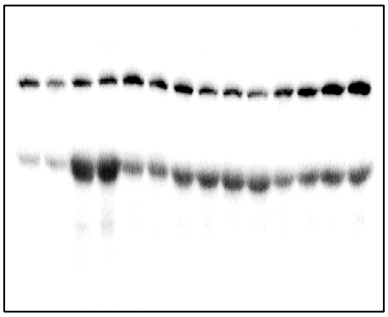

– 95kDa

- **The following were referred to western blot results in Figure 4**

NOX4

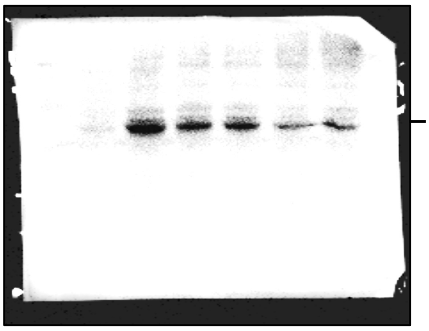

– 67kDa

p-ERK

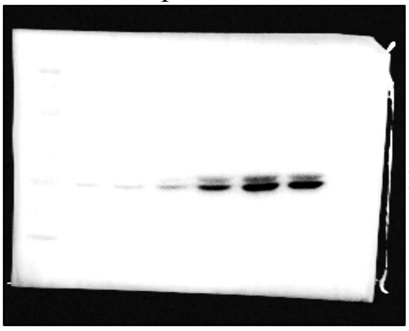

– 44kDa
– 42kDa

EEF2

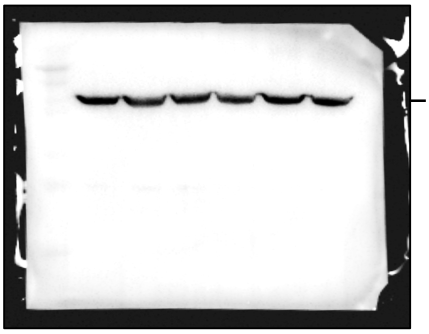

– 95kDa

ERK

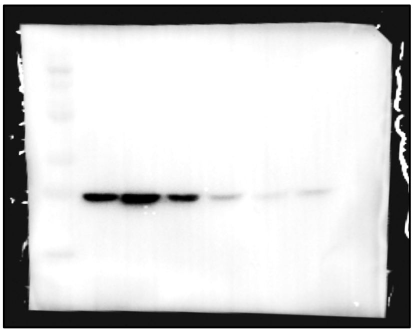

– 44kDa

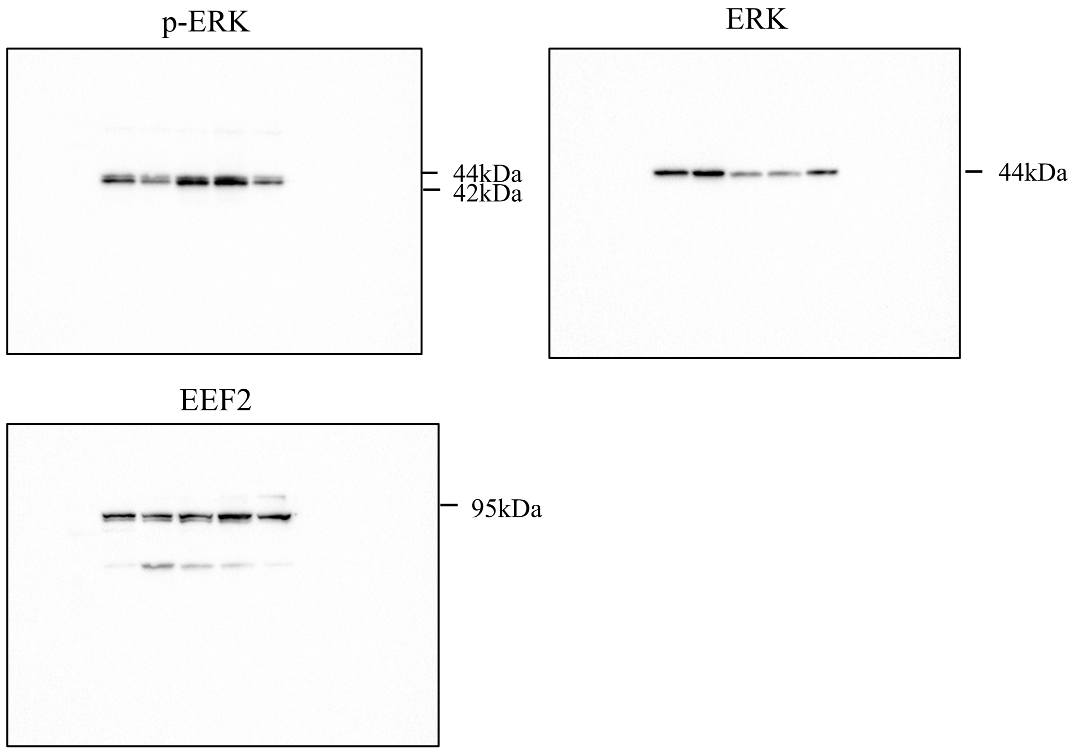

p-ERK
44kDa
42kDa
ERK
44kDa
EEF2
95kDa
